# Suppress to Survive—Implication of Plant Viruses in PTGS

**DOI:** 10.1007/s11105-014-0755-8

**Published:** 2014-07-09

**Authors:** Przemysław Wieczorek, Aleksandra Obrępalska-Stęplowska

**Affiliations:** Interdepartmental Laboratory of Molecular Biology, Institute of Plant Protection-National Research Institute, 20 Władysława Węgorka St, 60-318 Poznań, Poland

**Keywords:** Plant viruses, Post-transcriptional gene silencing, Suppressors of PTGS, Plant defense, Counter-defense proteins

## Abstract

In higher plants, evolutionarily conserved processes playing an essential role during gene expression rely on small noncoding RNA molecules (sRNA). Within a wide range of sRNA-dependent cellular events, there is posttranscriptional gene silencing, the process that is activated in response to the presence of double-stranded RNAs (dsRNAs) in planta. The sequence-specific mechanism of silencing is based on RNase-mediated trimming of dsRNAs into translationally inactive short molecules. Viruses invading and replicating in host are also a source of dsRNAs and are recognized as such by cellular posttranscriptional silencing machinery leading to degradation of the pathogenic RNA. However, viruses are not totally defenseless. In parallel with evolving plant defense strategies, viruses have managed a wide range of multifunctional proteins that efficiently impede the posttranscriptional gene silencing. These viral counteracting factors are known as suppressors of RNA silencing. The aim of this review is to summarize the role and the mode of action of several functionally characterized RNA silencing suppressors encoded by RNA viruses directly involved in plant–pathogen interactions. Additionally, we point out that the widely diverse functions, structures, and modes of action of viral suppressors can be performed by different proteins, even in related viruses. All those adaptations have been evolved to achieve the same goal: to maximize the rate of viral genetic material replication by interrupting the evolutionary conserved plant defense mechanism of posttranscriptional gene silencing.

## General Overview of Posttranscriptional Gene Silencing Induced During Viral Infection

In eukaryotes, posttranscriptional gene-silencing (PTGS) plays a pivotal role in the regulation of gene expression during development (Sunkar 2012; Wienholds and Plasterk 2005), stress feedback (Ferguson 2011) or genome stability maintenance (van Wolfswinkel and Ketting 2010). It is also induced in response to an invasion of molecular parasites, such as viruses as well as other factors similar in structure and biological properties (viroids, satellite RNAs, defecting RNAs, and defecting-interfering RNAs) (Yang et al. 2011). Although no antibodies (that would maintain stable immunological memory against viruses) have been identified in plants so far, plants utilize PTGS to efficiently and specifically recognize and eliminate those molecular pathogens. Viruses—not possessing any redundant genetic cargo—take a great advantage of cellular biochemical machinery to replicate in infected host cells. Importantly, every type of plant viruses (DNA, RNA, single (ssRNA) or double stranded, of positive or negative polarity of their genome) has to overcome the RNA stage that constitutes a source of PTGS-inducing molecules (dsRNA) (Baulcombe 2004).

Four well-defined steps can be distinguished during PTGS: (1) detection of the dsRNAs, (2) generation and amplification of small interfering RNAs (siRNAs), (3) silencing of viral target gene, and finally, (4) spreading of the signal between plant cells and within the host through the vasculature (Chitwood and Timmermans 2010; Kalantidis et al. 2008). dsRNAs in the cytoplasm serve as strong signaling molecules recognized by the cellular nucleases that subsequently cleave dsRNA to short (21–24 nucleotides (nt)) fragments. The nucleases, known in plants as Dicer-like proteins (DCL), belong to ribonuclease III family and have a strong affinity toward dsRNA (Liu et al. 2009).

At this point, it is important to notice that in a cell, there is also a fraction of endogenously generated primary small noncoding RNAs (reviewed in details by Axtell 2013): natural antisense transcript siRNAs (nat-siRNA), trans-acting siRNAs (ta-siRNAs), and microRNAs (miRNAs). The latter are originated from specific genomic loci described as *MIR* genes (discussed by Rogers and Chen 2013; Zhang et al. 2006a). The *MIR* genes are transcribed by RNA polymerase II in a form of long structured hairpin-containing transcripts that are subsequently put under the several processing stages dependent on DCL nucleases and methylases, among others (Axtell et al. 2011; Rogers and Chen 2013).

Among four identified in *Arabidopsis thaliana* DCLs, DCL4 was found to be the most involved in processing of virus-derived siRNAs. For its biochemical activity, DCL4 requires a molecular partner encoded by host plant: dsRNA-binding protein 4 (DRB4) (Fukudome et al. 2011). Interestingly, it was indicated by Ding (2010) and Llave (2010) that within overall siRNA pool, the 22-nt viral siRNAs were produced by DCL2 in the presence of DCL4, and this fraction constituted <20 % of the total viral small RNAs population. DCL1 seems to have a lesser contribution to the process (Deleris et al. 2006; Llave 2010). In triple *dcl2 dcl3 dcl4 A. thaliana* mutant, low yet detectable level of virus-derived siRNAs was identified under infection of *Turnip mosaic virus*, suggesting that this DCL1 can have a minor function during antiviral response (Blevins et al. 2006; Bouche et al. 2006).

The siRNAs activate the next step of the silencing described generally as RNA-dependent RNA degradation. At this stage of PTGS, siRNAs are loaded into RNA-induced silencing complex (RISC) standing in the central position of the PTGS. The nucleolytic, slicing core of the RISC complex consists of, among others, Argonaute (AGO) protein (Baumberger and Baulcombe 2005; Parker 2010; Wang et al. 2009) which, when loaded with siRNAs, undergoes scanning of target transcripts (or viral RNAs) and recognizes only those complementary with the siRNA probe. A family of ten AGO proteins was identified in *Arabidopsis* (Vaucheret 2008), whereas 15 AGO genes were described in *Solanum lycopersicum* (Bai et al. 2012; Xian et al. 2013), and nine of AGO homologs were found in the de novo-sequenced *Nicotiana benthamiana* transcriptome (Nakasugi et al. 2013). In *A. thaliana* AGO1, AGO2, AGO5, and AGO7 can bind siRNA, thus taking part in antiviral defense (Qu et al. 2008; Takeda et al. 2008). However, it is presumed that mainly AGO1 plays an essential role in anti-viral defense in plants (Zhang et al. 2006b), and its function might be supported by AGO2 during this process (Harvey et al. 2011). According to the authors, the AGO2 compensates the antiviral function of AGO1 while the former is being inhibited in a presence of PTGS suppressor. Nevertheless, Scholthof et al. stated in 2011 that AGO2 from *N. benthamiana* (NbAGO2) plays the key and specific role in the anti-*Tomato bushy stunt virus* (TBSV) silencing (Scholthof et al. 2011). Antiviral importance of AGO2 was then indicated in *A. thaliana* infected with *Potato virus X* (PVX) (Jaubert et al. 2011) and *Turnip crinkle virus* (TCV) (Zhang et al. 2012b). Additionally, antiviral function of AGO4 was proposed during *Cucumber mosaic virus* (CMV) infection in *N. benthamiana* (Ye et al. 2009).

Once the target messenger RNA (mRNA; or viral RNA) is identified by programmed RISC, it is either cleaved by AGO or it can be translationally unreadable (Bartel 2004; Tolia and Joshua-Tor 2007). In fact, specific protein is no longer being produced.

Moreover, primary siRNAs derived from direct DCL-dependent dicing of the long dsRNA templates can promote accumulation of secondary fraction of siRNAs. This process is considered to be an amplification of PTGS signal mediated by plant RNA-dependent RNA polymerases (RDR) (Cuperus et al. 2010; Garcia-Ruiz et al. 2010; Wang et al. 2010; Wang et al. 2011) interacting with cellular suppressor of gene silencing 3 (SGS3) (Kumakura et al. 2009).

## Origin of Virus-Derived siRNAs

Two major RNA elements of PTGS are required to sequence-specific inhibition of viral RNAs expression: inducer of the PTGS—dsRNA and effector molecules—siRNAs. Virus replication is restricted to specific cell compartments (den Boon and Ahlquist 2010). This spatial separation protects viral genome, at least partly, from the exposition to cellular DCLs and nucleolytic degradation. However, accumulation of dsRNA molecules, at least temporarily, was observed during multiplication of genomic RNA and transcription of subgenomic viral mRNAs. It is also strongly assumed that viral siRNAs might be derived from intramolecular fold-back structures within viral genome. This is also supported by data from deep-sequencing experiments which shows that short 20–24 nt RNAs were not distributed evenly within viral genomic RNA, and presence of characteristic sRNA hotspots (loci characterized with higher distribution of specific sRNA) was frequently observed (Aregger et al. 2012; Kalischuk et al. 2013; Mitter et al. 2013).

## Approaches in the RNA Silencing Suppressors Identification

The basic experimental identification of RNA-silencing suppressors (RSSs) was described previously by various authors (Johansen and Carrington 2001; Li and Ding 2006; Ma et al. 2009) and reviewed by Vargason et al. (2013). Three major components of the classic patch assay are required: (1) a gene to be silenced, (2) the inducer of its silencing, and (3) the studied viral protein—the putative suppressor of PTGS (Johansen and Carrington 2001). Briefly, in a presence of PTGS inducer—for instance hairpin double-stranded RNA—its target complementary mRNA (encoding a reporter gene) is efficiently silenced. However, co-expression of a PTGS suppressor stabilizes the mRNA level and the reporter gene activity. This is manifested by the intact level of reporter mRNA and barely detectable amounts of corresponding siRNAs. Conversely, lack of the suppressor leads to mRNA degradation and accumulation of siRNAs.

Moreover, silencing can occur transiently as well. This is possible because locally induced silencing, in majority of cases, is followed by systemic spread of the PTGS-inducing signals within the whole plant (Voinnet et al. 1998), which can be verified by measuring transgene expression in systemic leaves. Long-distance movement of PTGS-inducing siRNAs was also proved by an elegant experiment based on a grafting assay, where the silencing signal had been spreading from silenced rootstock into intact scion expressing marker gene (Kalantidis 2004; Mallory et al. 2001). In result, expression of reporter marker was silenced both in the rootstock and the scion. It was supposed and proved that expression of the RSS should restore activity of the silenced transgene. Similar effect is observed when RSS is expressed in plants with stably silenced reporter gene—the suppressor efficiently reverses induced PTGS, and as a result, expression of the marker is restored.

Delivery of the RSS can be done in several ways: locally (for instance by agroinfiltration), transgenetically (by transformation) (Yu et al. 2006), via crossing the silenced plant with RSS-expressing one, or by means of virus-based expressing vectors (Cao et al. 2005; Niu et al. 2009). The virus-based expressing approach was used to identify PTGS-suppressing activity of P29—a papain-like protease from *Cryphonectria* hypovirus 1 (Segers et al. 2006) or βC1 from *Ageratum yellow vein virus* (Sharma et al. 2010). However, influence of the expressing vector itself cannot be omitted, and therefore, data delivered from such an approach must be interpreted carefully.

Most research papers describe the *Agrobacterium*-based transient expression tools as sufficient to verify preliminarily suppressing activity of analyzed viral proteins. This is very convenient, especially when an easily detectable, efficient, and time-saving reporter gene, for instance green fluorescent protein (GFP), is used.

To determine the exact functional abilities of RSS, further analyses are required. Assessment of RSS affinity towards siRNA or long dsRNA can be done by electrophoretic mobility shift assays (EMSA), immunoprecipitation (IP), or co-immonoprecipitation (co-IP) of the ribonucleoprotein complexes. This allows to determine the siRNA–RSS affinity and the specificity of their interactions that, together with experiments based on site-directed mutagenesis of the RSS, can provide essential information on its biological role.

In papers published by Pantaleo et al. (2007) and Csorba et al. (2010), the authors proposed a simple yet informative system, adopted and modified from a technique described previously (Parizotto et al. 2004), useful for the identification of interactions between RSS and specific miRNAs or other components of the PTGS pathway. The sensor system utilizes the in vivo transcribed engineered GFP-coding mRNA possessing a complementary miRNA target site incorporated within 3′UTR of the reporter gene. In a presence of specific miRNA, molecule expression of the reporter gene is inhibited only if the sensor bears target site recognizable by the short RNA. Only specific interaction between RSS and miRNA can abolish the inhibitory potential of the short RNA, and as a result, enable expression of the reporter gene. Such a strategy, together with co-expression of candidate RSS, is an ideal tool for revealing, for instance, the miRNA turnover in the presence of a PTGS suppressor.

## Mechanisms of Suppression of Virus-Induced PTGS

After the delivery of viral genetic material into the plant, PTGS machinery recognizes the pathogenic RNA, which in turn leads to its degradation. At this very stage, only immediate viral response to PTGS would enable the virus to spread systemically. In fact, this virus counteraction might be based on: (1) binding of the long dsRNA and their protection from the subsequent DCL processing, (2) sequestration and/or degradation of siRNAs, (3) inactivation of functional RISCs, (4) inhibition of short- and long-distance spread of the silencing signal. Indeed, viral suppressors can interrupt the PTGS utilizing at least one of the mentioned mechanisms.

**Fig. 1:**
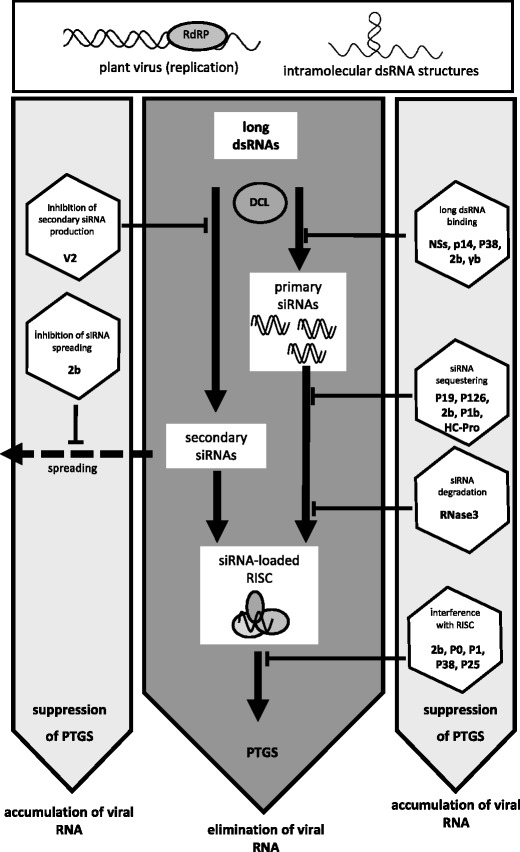
Examples of plant virus-encoded RNA silencing suppressors and points of their action. Double-stranded RNA (dsRNA) structures can be formed during virus infection as replication intermediates or can be generated through intramolecular base pairing within genomic (or subgenomic) RNA strands (*upper box*). The dsRNAs are then recognized by Dicer-like proteins (*DCL*) initiating posttranscriptional gene silencing (*PTGS*) pathway (*middle dark gray-shaded panel*). Primary and secondary small interfering RNAs (*siRNA*) are produced and incorporated subsequently into RNA-induced silencing complex (*RISC*), leading to nucleolytic elimination of viral RNA. Additionally, siRNA are transported to surrounding cells what is considered as a spreading of the silencing signal (*dashed arrow*). However, PTGS suppressors encoded by viruses interfere with the plant defense mechanisms by interfering with different stages of the PTGS (*left and right side light gray-shaded panels*) and this leads to accumulation of viral genetic material and its expression in infected tissues. Examples of particular RNA silencing suppressors are indicated in *hexagons* pointing on their proposed silencing modes and points of action during the PTGS

Several examples of known RSSs were listed in Table 1 and indicated in Fig. 1. Importantly, the suppressing activity of viral proteins is shared with their other biological functions essential during virus replication cycle. This correlates with general genetic abilities of viruses: to encode only absolutely essential genes by relatively small genomes. For instance, potyviral HC-Pro is both a RSS and a helper component of viral proteinase required for virus transmission and systemic movement (Sáenz et al. 2002), whereas P38 of TCV inhibits PTGS and constitutes a component unit of viral capsid (Azevedo et al. 2010). Similarly, structural function of *Tomato chlorosis virus* coat protein (CP) and P6 virion protein of *Rice yellow stunt rhabdovirus* is shared with its PTGS-suppressing activity (Cañizares et al. 2013; Guo et al. 2013). More interestingly, *Tobacco mosaic virus* P126 protein contains three domains: N-terminal methyltransferase (MET), two nonconserved regions (NONI and NONII), and helicase (HEL), each exhibiting independently both local and systemic PTGS-suppressing activities (Wang et al. 2012).

**Table 1:** Examples of viral suppressors of PTGS

Virus name (acronym)	Identified RSS	Biological function	Proposed mechanism of PTGS suppression	References
*Tobacco mosaic virus* (TMV)	P126	Multidomain protein with helicase and methyltransferase activities; RNA genome replication, viral cell-to-cell movement	HEL, MET, and NONII domains with RSS activity, binds siRNA in size-selective manner	Wang et al. (2012)
*Tomato aspermy virus* (TAV)	2b	Homolog of CMV 2b	Sequestrates siRNAs, binds sRNAs in length-specific and sequence-independent manner	Chen et al. (2008)
*Cucumber mosaic virus* (CMV)	2b	Symptom induction, virulence determinant, host-specific virus accumulation	Sequestrates long and short dsRNAs, interacts with AGO1, interacts with AGO4	Zhang et al. (2006b) and Gonzalez et al. (2012)
*Carnation Italian ringspot virus* (CIRV)	P19	Pathogenicity determinant, symptom-severity modulator	Sequestrates siRNAs in sequence-independent manner	Vargason et al. (2003)
*Potato virus X* (PVX)	P25 (TGBp1)	Cell-to-cell movement	Interacts with AGO1 and mediates its proteasome-dependent degradation	Chiu et al. (2010)
*Turnip crinkle virus* (TCV)	P38	Coat protein, virion structure	Binds and inhibits AGO1 through the GW motif	Azevedo et al. (2010)
*Barley stripe mosaic virus* (BSMV)	Γb	Pathogenicity determinant, viral long-distance movement, genome amplification	Binds ds-sRNA in size-selective manner	Yelina et al. (2002) and Mérai et al. (2006)
*Citrus leaf bloth virus* (CLBV)	MP	Week PTGS suppression, local function, does not inhibit cell-to-cell and long movement of silencing signal	Not described	Renovell et al. (2012)
*Sweet potato mild mottle virus* (SPMMV)	P1	Serine protease, processing of viral polyprotein	Binds to argonaute and inhibits RNA-induced silencing complex activity	Giner et al. (2010)
*Cucumber vein yellowing virus* (CVYV)	P1b	Serine protease, processing of viral polyprotein	Binds 21-nucleotide (nt) sRNAs	Valli et al. (2011)
*Turnip mosaic virus* (TuMV), *Potato virus Y* (PVY)	HC-Pro	Cysteine protease, viral polyprotein processing, systemic movement, pathogenicity determinant	Binds short RNAs, interacts with proteasome antiviral activity	Jin et al. (2007) and Chapman et al. (2004)

Biological function as well as proposed implication in the PTGS was indicated

RNA silencing is a common process described across all kingdoms, in plant and animal systems, and consequently, suppression of it was described for plant and animal viruses. Importantly, plant viral suppressors of PTGS retain their biological function in animals, and vice versa. For instance, VP3 protein of avian *Infectious bursal disease virus* efficiently suppresses PTGS in plants, and can functionally replace HC-Pro-silencing suppressor of plant *Plum pox virus* (Valli et al. 2012). Maliogka et al. (2012) used the same engineered *Plum pox virus* potyviral background to test suppressor activity of other unrelated RSS from both plant (P1b from *Cucumber vein yellowing virus*, P19 from TBSV) and animal viruses (influenza A virus NS1). Conversely, Young et al. (2012) have shown that related viruses from *Potyviridae* family can suppress PTGS using different proteins, and possibly, three different suppressing pathways: P1 (tritimoviruses), P1 or P1b (ipomoviruses), and HC-Pro (potyviruses).

As it will be described in the following paragraphs, the viral RSSs represent a wide biological diversity in structure, mode of action, as well as their impact on host plant. However, despite the fact that knowledge from the field of RSS is constantly verified and updated, it still seems that the fundamental mechanism of this particular virus–host interaction is yet to be discovered. New experimental data broadens and deepens the view on the RSS functional complexity. It puts rather significant emphasis on the occurrence of several levels of plant defense and virus counter-defense relationships, at least at the PTGS level (reviewed on an example of 2b of CMV by Masuta and Shimura 2013 or tombusvirus P19 by Várallyay et al. 2014).

### Binding of Long dsRNAs: Inhibition of the Dicing Steps

Long dsRNAs formed both locally within ssRNA segments, as well as during viral replication or produced de novo by host RDR activate PGTS machinery. Therefore, dsRNA protection could be one of the initial steps, where suppressors guarding the viral RNAs from their DCL-dependent degradation operate. NSs suppressor of *Tomato spotted wilt virus* is an example of such a mechanism. The protein can efficiently bind both long and short dsRNAs (Schnettler et al. 2010) indicating that NSs activity might be situated up- or downstream of DCL dicing. Moreover, it was shown recently that PTGS-suppressing domain is located—together with hypersensitive response-triggering avirulence determinant of the *Tomato spotted wilt virus*—within N-terminal part of NSs (de Ronde et al. 2014). Similar function of NSs was described also for other tospoviruses: *Impatiens necrotic spot virus* and *Groundnut ringspot virus* (Schnettler et al. 2010). Contrarily, another member of the family, *Tomato yellow ring virus*, expresses the NS that binds only short dsRNAs. The affinity of NSs suppressors of some tospoviruses to long dsRNA is not clearly understood, yet it is assumed that it might result from possible binding of the NS to hairpin elements from 3′-untranslated region of viral transcripts. This RNA–protein interaction is postulated to enhance translation of virus-derived transcripts (Geerts-Dimitriadou et al. 2012).

Binding of long dsRNAs by RSS is not limited only to tospoviruses. CP of carmovirus TCV and p14 of *Pothos latent virus* have an affinity to long dsRNA as well (Mérai et al. 2005, 2006).

The suppressors of the PTGS were also identified among proteins with inhibitory properties towards DCL’s function—thus impairing the dicing of dsRNAs. Experimental data obtained by Cao et al. (2010) indicated that strong PTGS suppressor P38 encoded by TCV efficiently suppressed the DCL activity in *A. thaliana*. Interaction between DCL4 and *Cauliflower mosaic virus* P6 was reported to play an important role in suppression of PTGS (Haas et al. 2008).

### Separation of Virus-Specific siRNAs from PTGS Machinery

#### siRNA Sequestration

The generalization that RSS-mediated suppression of PTGS would be based on binding of siRNAs was made by Lakatos et al. (2006). Indeed, this strategy seems to be represented by substantial number of known RSSs. For instance, tombusvirus P19 protein is a well-characterized RSS, whose function was analyzed in both plant and animal systems (Liu et al. 2012; Vargason et al. 2003). Biological activity of this suppressor depends on the generation of a stable tail-to-tail homodimer structure, which determines binding to siRNAs. The general mode of action of this RSS is based on size-specific binding of dsRNAs in a sequence-independent manner, probably by means of direct RNA binding by positively charged amino acids localized on P19 surface (Liu et al. 2012). Additionally, the affinity of the P19 was shown to be siRNAs length-dependent with the highest values for 21 nt siRNAs (Vargason et al. 2003).

Having taken into consideration the affinity of known RSS to small RNAs, there was also a need to analyze interactions between the suppressors and microRNAs. Schnettler et al. (2010) published a paper in which the authors studied affinity of tospovirus NSs protein toward various short RNAs, including miRNAs. The authors concluded that tospoviruses interfere with PTGS by sequestering siRNAs and miRNAs molecules before they are loaded into their respective RNA-induced silencing complexes.

#### siRNA Degradation

As it was mentioned previously, sequestration of virus-derived siRNAs by RSS is an efficient way to suppress PTGS. However, Cuellar et al. (2009) have shown another mechanism inhibiting the process. *Sweet potato chlorotic stunt virus* (Cuellar et al. 2009) encodes RNase3 that binds and cleaves siRNAs into 14 bp products that no longer can activate the RISC targeting to slice *Sweet potato chlorotic stunt virus* RNA. Endonucleolytic activity of the RNase3 was supported by Mn^2+^ and was most efficient at pH 7.5 (long dsRNA), pH 8.5 (long and small dsRNA), and pH 8 (Weinheimer et al. 2014).

#### Inhibition of siRNA Transport

Antiviral defense based on PTGS requires spreading of the silencing signal from cell to cell, and finally, within the whole plant. Therefore, suppression strategy based on inhibition of siRNAs transport might be a mechanism allowing viruses to overcome the host defense. For instance, 2b protein of CMV can inhibit spreading of the silencing signal (Guo and Ding 2002). Molecular basis of siRNA binding by 2b suppressor was examined in crystallographic studies of RSS encoded by another cucumovirus, *Tomato aspermy virus.* Studies performed by Chen et al. (2008) indicated that *Tomato aspermy virus* 2b recognizes siRNAs by the pair of “hook-like” structures that allow the protein to bind to siRNA duplex and long dsRNA in a length-independent manner*.*

#### Secondary siRNAs Synthesis Inhibition

After recognition of dsRNA by DCL, the primary pool of siRNA is being produced. The resulting siRNAs are then subsequently loaded into the RISC leading to the production of cleaved, aberrant RNAs. Such RNAs are recognized by cellular RDRs which produce another pool of long dsRNAs out of which secondary short RNAs are diced. This stage is dependent on the interaction of two protein partners: SGS3/RDR6 (Kumakura et al. 2009; Mourrain et al. 2000; Peragine et al. 2004). SGS3 is a dsRNA-binding protein that shares specificity to the substrate with *Tomato yellow leaf curl virus* V2 (Fukunaga and Doudna 2009), which interacts directly with SGS3 *in planta* (Glick et al. 2008). The V2 outcompetes SGS3 in binding, for instance, viral dsRNA. As a result, production of the virus-derived siRNAs is inhibited (Fukunaga and Doudna 2009). Interestingly, V2 of *Tomato yellow leaf curl China virus*, another member of *Begomovirus*, possesses completely different mode of PTGS suppression: generally the protein does not interact with SGS3 and rather sequestrates siRNAs (Zhang et al. 2012a).

### Alteration of Effector Complex

One of the possible mechanisms of PTGS suppression is inactivation of the “slicer” function of the RISC core protein. As it was mentioned previously, RISC is the effector in process of the PTGS, and AGO proteins are responsible for its nucleolytic activity. In the case of *A. thaliana,* AGO1 protein plays an essential role in degradation of the target RNA. Several RSSes were found to have a direct impact on effector component of RISC. Well characterized protein 2b encoded by CMV can interact with AGO1 loaded with siRNA, and inhibit its cleavage properties. Interestingly, it was reported by Hamera et al. (2012) that 2b of CMV also interacts with the host plant AGO4 protein by recognition of its PAZ and PIWI domains counteracting AGO4-related functions during RNA-dependent DNA methylation. AGO4-derived 24-nt siRNAs were found in 2b-sRNAs complexes, indicating that the RSS recognized the small RNAs specifically. However, as Duan et al. (2012) showed, within CMV 2b there are two separate N- and C-terminal domains responsible for dsRNA binding and AGO interaction, respectively. The siRNA–AGO–2b interactions have been revealed to be more sophisticated. The authors showed that 2b-mediated suppression of PTGS in *A. thaliana* is directed by 2b-siRNA binding, and is supported by rather than dependent on 2b–AGO interactions.

Baumberger and others showed (Baumberger et al. 2007; Bortolamiol et al. 2007) that a *Polerovirus*-encoded F-box motif of protein P0 mediates AGO1 for proteolysis and degradation. Moreover, when another member of poleroviruses, *Sugarcane yellow leaf virus*, has been analyzed, it displayed a surprisingly different mechanism of P0-dependent PTGS suppression. Unlike proteins P0^BW^ and P0^CA^, encoded by *Beet western yellow virus* and *Cucurbit aphid-borne yellow virus*, respectively, the *Sugarcane yellow leaf virus* P0 can suppress local silencing as well as systemic spread of silencing signal.

Regarding the importance of the AGO during PTGS, its function is supported by plant-encoded proteins which interact with AGO via GW/WG (glycine tryptophan/tryptophan glycine) motifs. Therefore, the question arose whether viral RSS proteins containing GW/WG motifs can inhibit PTGS via interaction with AGO. It was shown that P1 protein of *Sweet potato mild mottle virus* possesses three GW/WG motifs (Giner et al. 2010) that can mimic host proteins binding to AGO1-loaded RISC and thus counteract plant RNA silencing effectors. Interestingly, it was experimentally shown that *Sweet potato feathery mottle virus* P1, a GW/WG-lacking homologue of the *Sweet potato mild mottle virus* P1 does not possess any PTGS-suppressing activity. Two GW/WG motifs introduced experimentally into P1 converted the RSS-inactive protein into a functionally active one (Szabo et al. 2012). In the case of TCV, its multifunctional P38 was reported to compete with cellular GW/WG-containing proteins resulting in suppression of antiviral defense.

## RSS Involvement in Plant Pathogenicity

The mode of action of known viral RSSs is sophisticated: it targets very sensitive plant metabolic pathways and disrupts homeostasis of cellular regulatory signals based on distribution of small regulatory RNAs. Therefore, it is not surprising that the occurrence of RSS in plant cells might be connected with macroscopic changes manifested with disease-like symptoms, for instance leaves malformation, stem stunting or local and necrotic lesions.

In a paper of Siddiqui et al. (2008), the authors analyzed phenotypic effects developed in *N. benthamiana* and *N. tabacum* stably transformed with seven viral-silencing suppressors originated from different virus genera: P1 of *Rice yellow mottle virus* (*Sobemovirus*), P1 of *Cocksfoot mottle virus* (*Sobemovirus*), P19 of TBSV (*Tombusvirus*), P25 of PVX (*Potexvirus*), HC-Pro of *Potato virus Y* (*Potyvirus*), 2b of CMV (*Cucumovirus*), and AC2 of *African cassava mosaic virus* (*Begomovirus*). The authors concluded that a wide range of effects manifested differently upon expression of particular RSSs with regard to transformed tobacco species. Going further, Soitamo and colleagues asked whether and how the phenotypic effect that resulted from RSS expression in plant is connected with transcriptome and proteome changes (Soitamo et al. 2011). Using both high-throughput transcriptomic (microarray) and proteomic (2-DE) approaches, the authors have shown that expression of PVY HC-Pro in transgenic plants upregulated, among others, defense-, stress-, photosynthesis-related genes. The same authors investigated cellular effect of AC2-silencing suppressor of *African cassava mosaic virus* expressed in transgenic tobacco (Soitamo et al. 2012). They found 1118 and 251 transcripts altered in levels in leaves and flowers, respectively. Most upregulated transcripts were associated with signaling, cell wall modifications, and stress response. When comparing the levels of altered transcripts in plants expressing HC-Pro and AC2, the authors concluded that ca. 500 and 300 transcripts were up- and downregulated, respectively. Additional proteomic profile of *N. benthamiana* was established by Carmo et al. (2013), who tested an influence of the AC2 of *Tomato chlorotic mottle virus* expressed heterologously in tobacco from PVX vector. The authors showed that AC2 disrupts a wide range of cellular mechanisms related with photosynthesis, defense or oxidative stress response, which is consistent, at least in part, with the data presented by Soitamo et al. (2012).

Going further, it was interesting whether viral RSS can modulate AGO expression and miRNA-dependent regulation. As it was shown by Várallyay et al. (2010), plant viruses induce miR168 that negatively regulates antiviral AGO1. Subsequently, Várallyay and Havelda (2013) postulated that specifically RSS-induced over-accumulation of miR168 might play an essential role in disease symptom development in virus infected plants. Here, the RSS would be a precise trigger that induces miR168. Indeed, using *Agrobacterium*-mediated transient expression assay the authors expressed different unrelated RSS (P122 of crucifer-infecting *Tobamovirus*, P19 of *Cymbidium ringspot virus*, P38 of TCV, HC-Pro of *Tobacco etch virus,* and 2b of CMV) in *A. thaliana,* and revealed over-accumulation of miR168 and downregulation of AGO1. Interestingly, it was shown that this miR168 upregulation is not dependent on P19 siRNA-binding abilities (Várallyay et al. 2014). Still, P19-3M—the P19 mutant that cannot bind siRNAs—regulates the levels of miR168 and AGO1. Additionally, although *Carnation Italian ringspot virus* carrying P19-3M variant accumulates in *N. benthamiana* to lower level, it still causes intermediate severity symptoms in infected plants.

Taking into consideration the interactions between miRNAs and RSS, it was shown that viral suppressors of PTGS can interfere with miRNA-mediated silencing pathways leading to developmental defects (Chapman et al. 2004; Jay et al. 2011; Shen et al. 2012; Stav et al. 2010).

## Concluding Remarks

The most recent data indicate that RSSs encoded by plant viruses give an example of evolutionary molecular adaptation to the host antiviral defense. The proteins target the host plant PTGS pathways, which are responsible for efficient elimination of pathogenic RNAs from infected cells. Therefore, the dynamic equilibrium between the RSS and the PTGS components playing the crucial role in antiviral defense, determines the expansion rate of the pathogenic RNAs into the host. Additionally, even closely related viruses can have PTGS-suppressing proteins characterized by completely opposite mode of action. Moreover, no evident similarities have been found in neither protein sequence nor structure between known suppressors of PTGS. Going further, no conserved mechanisms of RSS action were noted, even though the cross-kingdom biological activity of PTGS suppressors was described. A common feature of RSS is their multifunctional character that manifests during virus replication. Some authors, however, suggest that the GW/WG motifs, as well as RNA-binding domains or positively charged amino acids localized within particular viral proteins, can predispose them to act as RSS (Bivalkar-Mehla et al. 2011). However, it is not a general rule. Thus, the identification of novel suppressors of PTGS needs to be always carried out and verified using a variety of experimental assays.

The knowledge of mechanisms of PTGS and its suppression is useful not only in a research on molecular biology of viruses. PTGS is frequently activated during transgenesis and manifests with lowered efficiency of transgene expression. Therefore, the appearance of a strong RSS can elevate the production efficiency of the recombinant protein (Gao et al. 2013; Garabagi et al. 2012; Haikonen et al. 2013; reviewed by Saunders and Lomonossoff 2013; Sun et al. 2011). However, in transgenic plants stably expressing RSS, unfavorable harmful developmental effects were observed. Saxena et al. (2011) overcame this by using modified p19 protein in the stable expression experiments. It was indicated that mutated p19—p19/R43W—can serve as a RSS after it was used in the following experimental cases: in transgenic expression of p19/R43W in *N. benthamiana,* in co-expression assay (with GFP), and in CPMV (*Cowpea mosaic virus*) expression system (Saxena et al. 2011).

Conversely, virus-based expression vectors seem to be alternative for genetically modified organisms and the cheapest platform for synthesis of heterologous proteins in plants (Hefferon 2012; Roy et al. 2011; Yusibov et al. 2013). The high level of proteins production and considerable yield obtained from transiently transformed plants, that in fact are the cheapest source of biomass, and this speaks for using the viral expression vectors. Engineering of disarmed viral vectors that possess inactivated pathogenicity determinant(s) expressed together with a strong RSS can be considered as a very efficient tool for the production of vaccines and therapeutic proteins in plants (as reviewed by Cañizares et al. 2005).

Usefulness of RSS in biotechnology is not confined only to plant engineering. Cheng et al. (2011) found tombusviral p19 suppressor as a promising tool in the analysis of human microRNAs function. In referred study, authors showed that mutational variants of p19 protein, differing with single amino acid residue localized within its binding surface, can greatly increase affinity for miR122 without altering p19-siRNA interactions. Thus, they postulate that p19 variants can be engineered to enhance their affinity toward specific small RNAs that differ in locations of base-pair mismatches (p19s with different binding surface variants can bind various miRNAs). Schuck et al. (2013) described recapitulated AGO/RISC in vitro system that might be used as a valuable tool in studying individual components of antiviral PTGS-based defense in plants.

In summary, here we gave a brief overview on functional diversity and complexity of viral PTGS suppressors and the functions they play in plants under virus infection. However, the stream of newly described data delivered from experiments that are being constantly performed in the area of RSS, will be putting a new light on viral suppressors, the mechanism they utilize and molecular interactions during pathogenesis. In the context of the newly described results, the previously speculated molecular interactions can be interpreted differently, and in the course of time, novel biochemical and functional abilities of RSS will be characterized. This, in turn, will enrich the general knowledge about molecular biology of viruses.

## Abbreviations

AGO: Argonaute proteins
DCL: Dicer-like proteins
dsRNA: Double-stranded RNA
miRNA: MicroRNA
PTGS: Posttranscriptional gene silencing
RISC: RNA-induced silencing complex
RSS: Suppressor of RNA silencing
sRNA: Short RNA
siRNA: Small interfering RNA
